# Differences between Active and Semi-Active Students Regarding the Parameters of Body Composition Using Bioimpedance and Magnetic Bioresonance Technologies

**DOI:** 10.3390/ijerph18157906

**Published:** 2021-07-26

**Authors:** Dana Badau, Adela Badau, Cristian Trambitas, Dia Trambitas-Miron, Raluca Moraru, Alexandru Antoniu Stan, Bogdan Marian Oancea, Ioan Turcu, Emilia Florina Grosu, Vlad Teodor Grosu, Lucia Georgeta Daina, Cristian Marius Daina, Corina Lacramioara Suteu, Liviu Moraru

**Affiliations:** 1 “Petru Maior” Faculty of Sciences and Letters, “George Emil Palade” University of Medicine, Pharmacy, Sciences and Technology, 540142 Targu Mures, Romania; dana.badau@umfst.ro; 2 Faculty of Medicine, “George Emil Palade” University of Medicine, Pharmacy, Sciences and Technology, 540142 Targu Mures, Romania; Dia.Trambitas-Miron@umfst.ro (D.T.-M.); raluca.moraru@umfst.ro (R.M.); alexandruastan@gmail.com (A.A.S.); liviu.moraru@umfst.ro (L.M.); 3 Faculty of Physical Education and Mountain Sports, Transilvania University, 500068 Brasov, Romania; bogdan.oancea@unitbv.ro (B.M.O.); ioan.turcu@unitbv.ro (I.T.); 4 Faculty of Physical Education and Sports, “Babes Bolyai” University, 540142 Cluj-Napoca, Romania; emiliaflorina.grosu@gmail.com; 5 Faculty of Automotive Mechatronics and Mechanical Engineering, Technical University of Cluj-Napoca, 400114 Cluj-Napoca, Romania; vlad.grosu@mdm.utcluj.ro; 6 Faculty of Medicine and Pharmacy, University of Oradea, 410087 Oradea, Romania; lucidaina@gmail.com (L.G.D.); cristi_daina@yahoo.co.uk (C.M.D.); suteucorina@gmail.com (C.L.S.)

**Keywords:** the obesity parameters, physical activity, active and semiactiv students, bioimpedance, magnetic bioresonance, health

## Abstract

The aim of the study was to identify differences in obesity-related parameters between active sports students and semi-active or sedentary students, differentiated by sex, in order to optimize health. The study sample included 286 students, of which the male experimental sample consisted of 86 active sports students, age X ± SD 21.25 ± 0.32 years; height X ± SD 181.08 ± 3.52 cm; control group consisting of 89 semi-active students aged X ± SD 21.07 ± 0.1.13 years; height X ± SD 182.11 ± 1.32. The female experimental sample includes 57 active sports students, age X ± SD 21.02 ± 0.92 years; height X ± SD 167.48 ± 1.34 cm; the control group includes 54 semi-active students aged X ± SD 21.57 ± 0.1.98 years; height X ± SD 168.42 ± 1.76. The study used a thalliometer, Tanita Health Ware software and Quantum Resonance Magnetic Analyzer equipment to investigate height (cm), Body Mass Index (BMI), muscle mass (kg, %), as well as the obesity analysis report, and componential analysis of body and nourishment. The differences registered between the samples of active and semi-active sports subjects were predominantly statistically significant for *p* < 0.05. The differences registered between the samples of active and semi-active sports subjects were predominantly statistically significant for *p* < 0.05. The most important parameters regarding obesity and body composition that registered significant differences between the two male groups were in favor of the group of active athletes: triglyceride content of abnormal coefficient 0.844 (CI95% 0.590–1.099), abnormal lipid metabolism coefficient 0.798 (CI95% 1.091–0.504), obesity degree of body (ODB %) 10.290 (CI95% 6.610–13.970), BMI 2.326 (CI95% 1.527–3.126), body fat (kg) 2.042 (CI95% 0.918–3.166), muscle volume (kg) 2.565 (CI95% 1.100–4.031), Lean body weight (kg) 2.841 (CI95% 5.265–0.418). In the case of female samples, the group of active sportswomen registered the biggest differences compared to the group of students who were significantly active in the parameters: abnormal lipid metabolism coefficient 1.063 (CI95% 1.380–0.746), triglyceride content of abnormal coefficient 0.807 (CI95% 0.437–1.178), obesity degree of body (ODB%) 8.082 (CI95% 2.983–13.181), BMI 2.285 (CI95% 1.247–3.324), body fat (kg) 2.586 (CI95% 0.905–4.267), muscle volume (kg) 2.570 (CI95% 0.154–4.985), lean body weight (kg) 4.118 (CI95% 1.160–7.077). The results of the study directly facilitate the understanding of the complexity of the impact of obesity on multiple parameters of body composition and health.

## 1. Introduction

Obesity at all ages is a globalized health problem with a major impact on quality of life [1,2,3]. Obesity management is a major desideratum of current health policies with a major impact on health and lifestyle. Obesity management, with major implications for optimizing health, is closely linked to the level of weekly physical activity, the quality and quantity of the diet and the approach to a proactive lifestyle. Health is dependent on a multitude of factors (physiological, somatic, mental, environmental, social, etc.), being a multidimensional concept aimed at the ability of social integrity and the environment, the dimensions of fitness, well-being, risk factors, etc. [4,5,6,7,8,9].

Recent studies have highlighted the major risk of lack of exercise in correlation with the increased incidence of obesity in cardiovascular disease, various cancers, diabetes and COVID-19 [10,11,12,13,14]. Obesity is considered a chronic disease that is caused by a number of major risk factors for human health, including genetic factor, hormonal balance, body thermogenic capacity, sensitivity of the nerve nucleus that regulates appetite or need for food, habits and behaviors related to unbalanced diet and sedentary lifestyle [15,16,17,18].

While the body mass index is the most researched obesity indicator [19], it is not sufficient to get a complete picture. Instead, a larger palette of factors should be considered while researching obesity, including phenotype, muscle mass, fat tissue level and its location, metabolism, triglyceride levels, and others [19,20,21]. An integrated/complete obesity study should take into account the correlation of body, tissue and metabolic parameters in order to be accurate and relevant [22,23]. The BIA approach is based on the use of recently proposed regression equations that estimate the body cell mass (BCM) and the anthropometric variables as the obesity degree of body (ODB %) and body mass index (BMI) [24,25]. Bio-resonance is a holistic biophysical method based on the recording and then on the decoding of electromagnetic frequency waves generated by unhealthy organs. The waves are influenced by DNA damage and by changes in the body’s magnetic energy field [26,27]. The magnetic resonance concept uses magnetic fields created by high power magnets which force the body’s protons to align with that field. A radio frequency current is then passed through the patient’s body, causing the protons to rotate outside the equilibrium parameters, tensioning against the magnetic field and providing information that is useful for generating detailed images of the inside of the body [28,29]. The assessment of the parameters of body composition by BIA shows similar levels of agreement with standard reference methods and other field-based techniques. For TANITA devices, the studies provided a valid measure of body composition [30,31,32].

Magnetic bioresonance and bioimpedance technologies are considered an unconventional and noninvasive methods of health assessment, and their current application aims to quantitatively and qualitatively identify the parameters of body composition for inclusion in complex models with multiple components. Body composition assessment requires a separate approach to each parameter and studies show that, combining parameters that deviate to different levels of sports training does not allow for an accurate assessment of body composition [33]. In practice, three methods of evaluating body composition are used, namely direct, indirect, or double-indirect approaches [34,35,36,37]. BIA is included among the methods of double-indirect approaches [33], in which the evaluation process uses validated regression equations, with estimates derived from indirect methods. BIA is a non-invasive and unconventional method, among the advantages of the method is the fact that the cumulative accuracy is proportional to the number of repetitions of the tests, and among the disadvantages it is mentioned that it is not possible to determine the distribution of body adipose tissue [33]. The use of BIA in practice and research specific to sports activity has significantly increased in the last decade due to portability, time and cost efficiency. The appearance of different devices with complex technologies has facilitated the extension of the possibilities of evaluating the parameters of body composition, but the comparison of the values calculated with these devices has multiple limits due to the fact that their reliability and calibration are different; aspects of electrode positioning, body position, and those related to the level of sports training, nutritional status, testing periodization, etc. are different. BIA was designed for general population samples, and the use of these technologies on specific samples, such as those of athletes, can provide relevant quantitative and qualitative information on body composition parameters, but also some inaccuracies in assessing body composition parameters, requiring readjustment of formula calculation by taking into account the specifics of sports training [38,39,40].

The studies have addressed the issue of obesity [2,41,42], and ways to reduce it under the influence of regular exercise [43,44,45]. Numerous studies reveal that degree of physical activity is associated with increased incidence of overweight and obesity, with a major impact on all age categories [46,47,48]. Expert recommendations stipulate at least 150 min of moderate-intensity aerobic activity or 75 min of vigorous-intensity aerobic activity, combined with two training sessions for muscle toning [49,50]. Prescribing exercise programs is based on age, physical fitness objectives of each individual, health level, preferences and motivations for certain types of physical activities, availability of time, intensity of activities, etc. [51,52,53].

The aim of the study was to identify differences in obesity-related parameters between active sports students and semi-active or sedentary students, differentiated by sex, in order to optimize health. The novelty of our study is to highlight a wide range of parameters associated with obesity using magnetic bioresonance and bioimpedance technologies for data collection, as well as by comparing two samples of students with different physical activity indexes, differentiated by sex.

## 2. Materials and Methods

### 2.1. Experimental Design

The study took place between October and December 2019 on a sample of 286 volunteer students. The study aimed at using the following measuring devices and software: thalliometer for investigating height (cm); Tanita Health Ware—Software [54], for the evaluation of BMI and muscle mass (kg, %); the Quantum Resonance Magnetic Analyzer equipment [55], for the investigation of three categories of parameters, namely the obesity analysis report, componential analysis of the body, and nourishment. Magnetic bioresonance and bioimpedance technologies are non-invasive and unconventional methods that complement classical methods by facilitating the evaluation of health and implicitly the parameters of body composition in an easy manner with increased cost and time efficiency. Deviant body composition parameters identified with BIA are calculated using linear mathematical methods to determine intra- and extracellular resistance values [33,56], single and multiple frequency BIAs that allow for separation into bioelectrical resistance and bioelectrical reactance [33,57,58]. The evaluations were performed in the time interval 9–11, in similar conditions for all study subjects. Each subject was evaluated once, with technologies used in the study. The evaluation of each subject was conducted in the gym with qualified staff for the registration and use of evaluation technologies. All study subjects performed the tests before performing any physical effort. For this article, all authors contributed equally; all authors have an equal contribution to the publication with the first author, too.

The obesity analysis report focused on the following parameters: abnormal lipid metabolism coefficient, brown adipose tissue abnormalities coefficient, hyperinsulinemia coefficient, nucleus of the hypothalamus abnormal coefficient, triglyceride content of abnormal coefficient [58]. The Componential Analysis of Body included parameters obesity degree of body (ODB%), body mass index (BMI), body cell mass (BCM). The obesity degree of body = weight real/Weight (kg) standard ··100, where the standard body weight, according to the World Health Organization for males is (height (cm) −80) · 70%, and for females, (height (cm) −70) · 60%. [59,60,61]. The nourishment parameters were intracellular fluid (L), extracellular fluid (L), protein (kg), inorganic substance, body fat (kg), body moisture (kg), muscle volume (kg), lean body weight (kg), weight (kg). Where total fluid volume = intracellular fluid (L) + extracellular fluid (L); muscle mass = total volume of fluids + proteins (kg), usually muscle mass is 35–48% of the weight; lean body weight (kg = muscle mass + inorganic substances (kg); weight = solid body mass + adipose tissue (kg); skeletal muscle mass (kg) = [(Ht2 /R · 0.401) + (gender · 3.825) + (age · −0.071)] +15.102, where Ht is height (cm); R is BIA resistance in ohms—for gender, men 51 and women 50; age (years) [62].

### 2.2. Subjects

The study sample included 286 students, of which 175 (61.2%) were male and 111 (38.8%) were female. Subjects were divided according to gender into two groups: the experiment group consisting of active sports students and the control group consisting of non-sports, inactive or semi-active students. The experimental groups consisted of students of a Bachelor’s degree in Physical Education and Sports, and the experimental groups from the kinetotherapy and nutrition and dietetics programs at the “George Emil Palade” University of Medicine, Pharmacy, Science and Technology from Targu Mures. The male experimental sample consisted of 86 active sports students, age X ± SD 21.25 ± 0.32 years; height X ± SD 181.08 ± 3.52 cm; control group consisting of 89 semi-active students aged X ± SD 21.07 ± 0.1.13 years; height X ± SD 182.11 ± 1.32. The female experimental sample includes 57 active sports students, age X ± SD 21.02 ± 0.92 years; height X ± SD 167.48 ± 1.34 cm; the control group includes 54 semi-active students aged X ± SD 21.57 ± 0.1.98 years; height X ± SD 168.42 ± 1.76. The male sample of the study included 87% subjects with urban residence and 13% with rural residence, and the female sample of the study had 91% urban and 9% rural origin. Inclusion criteria for students in the experimental group: age 18–25, physical and sports practitioners at least 5 times a week, duration of physical activity at least 250 min per week, no history of health problems or injuries in the last 3 months. Inclusion criteria for students in the control group: age 18–25 years, physical activity practitioners of maximum 100 min per week, with no history of health or injuries in the last 3 months.

### 2.3. Statistical Analyses

The research results were processed with SPSS 24 software, calculating statistical indicators: arithmetic mean (X), standard deviation (SD), Student’s test (*t*), average differences between sports and non-sports students (DX), Cohen’s (d) for effect size. Interpretation of effect size: small (0.2), medium (0.5) and large (0.8) [63]; the normality of distributions was assessed by using the Shapiro–Wilk test (S-W). Significance was set at *p* < 0.05 for all analyses. The specific reference values of the obesity analysis report are presented in Table 1. The total number of students was 765 and for this study we used 286; the calculated sample size must be a minimum of 211 subjects. The statistical power of study was (SP) 0.969 and the chosen level required was at least 0.8.

## 3. Results

Statistical processing of the results reveals that all differences between the two male groups were statistically significant in favor of the sports group, with the following exceptions for the brown adipose tissue abnormalities coefficient, the hyperinsulinemia coefficient and the nucleus of the hypothalamus abnormal coefficient. The results of the study groups are within the normal reference values, with the mention that those recorded by the group of athletes are closer to the lower limits of normality. The largest differences between the two groups, in favor of the group of athletes, were registered at the parameter the triglyceride content of abnormal coefficient 0.844, and the smallest difference at the parameter the nucleus of the hypothalamus abnormal coefficient −0.031. Cohen’s calculation for effect size showed that the following parameters had low and medium values between 0.2 and 0.5 for both samples: brown adipose tissue abnormalities coefficient hyperinsulinemia coefficient nucleus of the hypothalamus abnormal coefficient triglyceride content of abnormal coefficient; the abnormal lipid metabolism coefficient recorded a large effect of 0.82. The distribution of the results was normal, according to the S-W results which ranged between 0.711–0.918 (Table 2).

Regarding the statistical analysis of the parameters of the component analysis of the body, it reveals a strong statistical significance of the differences registered between the two male samples in favor of the group of athletes. At all parameters analyzed, the results recorded by the sample of athletes were lower than those of the group of non-athletes, which reflects the impact of exercise on body development. The distribution of the results was normal, according to the S-W results which ranged between 0.845–0.921 (Table 3). The most relevant differences were registered at the body mass index (BMI) where the group of athletes had 2.3267 less than the group of non-athletes, as well as at the degree of body obesity (ODB%) where the difference was 10.290. The analysis of the results of the statistical indicator Cohen’s for effect size for both male samples showed a large effect size of 0.89 and 0.82 for degree of body obesity (ODB%) and, respectively, body mass index (BMI); body cell mass (BCM) had 0.47 mean effect.

With the exception of the following parameters—the inorganic substance, protein (kg), the lean body weight (kg) and the weight (kg)—all the other analyzed parameters registered statistically significant differences between the group of athletes and the non-athletes. The most important parameters that registered significant differences between the two groups were: the body fat (kg), the muscle volume (kg) and the body moisture (kg); the group of athletes having much better values than that of non-athletes, revealing the importance of systematic exercise of physical exercise on body composition. The size of the size effect for both groups was large for parameters body fat 0.91, muscle volume 0.83, lean body weight 0.081 and weight 0.82; the other parameters recorded an average level of the Cohen’s for effect size statistical indicator. The distribution of the results was normal, according to the S-W results, which ranged between 0.769–0.923 (Table 4).

Statistical processing of the results reveals that all the differences between the two female groups were statistically significant in favor of the sports group, with one exception for the brown adipose tissue abnormalities coefficient. The results of the study groups are within the normal reference values, with the mention that those recorded by the group of athletes are closer to the lower limits of normality. The largest differences between the two groups, in favor of the sports group, were registered with the parameter abnormal lipid metabolism coefficient −1.063, and the smallest difference with the parameter the hyperinsulinemia coefficient −0.052. Cohen’s calculation for effect size showed that the following parameters recorded small and medium values between 0.2 and 0.5 for all parameters. The distribution of the results was normal, according to the S-W results, which ranged between 0.789–0.913 (Table 5).

The statistical processing of the parameters of the component analysis of body highlights a strong statistical significance of the differences between the two female samples, in favor of the group of athletes, except for body cell mass (BCM). At all parameters analyzed, the results recorded by the sample of athletes were lower than those of the group of non-athletes, which reflects the impact of exercise on body development. The most relevant differences were registered at the body mass index (BMI) where the group of athletes had 2285 less than the group of non-athletes, as well as at the obesity degree of body (ODB%) where the difference was 8082. The analysis of the results of both groups regarding the statistical indicator, Cohen’s, for effect showed large effect sizes of 0.84 and 0.87 for the obesity degree of body (ODB%) and, respectively, body mass index (BMI); body cell mass (BCM) had 0.38 mean effect. The distribution of the results was normal, according to the S-W results, which ranged between 0.815–0.911 (Table 6).

The results of the study on nourishment for the female groups show that only parameters: the body fat (kg), the muscle volume (kg) and the body moisture (kg) registered statistically significant differences between the two groups in favor of the sports group. The other analyzed parameters did not show significant differences as a result of systematically practicing physical exercises. The distribution of the results was normal, according to the S-W results, which ranged between 0.791–0.903 (Table 7).

## 4. Discussion

The analysis of the results contributes to the confirmation of the research purpose and facilitates the highlighting of significant differences in favor of the sample of active students, compared to the semi-active ones, analyzed and differentiated by sex. In all three categories of analyzed obesity parameters, namely the component analysis of the body, nourishment and the obesity analysis report, the female and male sample of sports students registered better values compared to those of semi-active students. The results of our study confirm previous studies that have analyzed the impact of physical activity on obesity [28,29,30,31], complementing the level of knowledge through the integrated analysis of three categories of parameters that analyze body obesity, namely the componential analysis of body, nourishment and the obesity analysis report.

The results of the male and female samples at the componential analysis of body reveal that the sample of sports students registered more optimal values compared to that of semi-active students for all parameters: obesity degree of body (ODB%), body mass index (BMI) and body cell mass (BCM). The athlete’s population sample recorded lower values of the degree of body obesity (ODB%) indicator compared to the non-athlete population in both sexes. This allows us to conclude that regular physical activity has a positive effect on real weight, relative to ideal weight (standard). The impact of physical training on optimizing body weight has been highlighted in many studies, focusing on the type of workouts and specific effort [64,65,66]. The results of the study are in agreement with previous studies that highlight the role of physical exercise, especially on body mass index (BMI), at different age groups [67,68,69], by sex [70,71] and depending on lifestyle [72,73].

In the obesity analysis report category, all two male groups, as well as the two female groups, recorded values that met the reference limits, but the sports student groups recorded better values towards the lower reference limits compared to the reference groups. semi-active student who recorded higher values, towards the upper reference limits. The registered level of triglyceride content of abnormal coefficient was above normal limits in both population samples and this can be correlated with the diet. We consider the increased values obtained for the athletes’ population sample are the result of using fat as an energy source. As such the results of our study are correlated with a study conducted on a sample of 235 student subjects (sports versus non-athletes) where the level of Triglyceride in both boys and girls was higher in athletes’ subjects [74]. In alignment with our results, other studies show that physical exercise can have a major influence in fighting obesity and lowering total cholesterol, but it does not influence the level of triglycerides, which increases due to physical activity [75].

Studies on body composition have shown the importance of keeping body weight and obesity-associated parameters within normal limits in order to optimize health [76,77,78]. The most relevant parameters in the nourishment category of the male and female sports student groups, compared to the similar groups of semi-active students, are highlighted by our study. We consider that they were registered with body fat and muscle volume parameters. The low level of body fat and the high level of muscle volume, compared to the average weight of the samples, highlight the major impact that regular physical activity has on body composition and, implicitly, on health. When analyzing the body fat level in correlation with weight (kg), we noticed that, in both sample populations, the level of adipose tissue was lower in athletes than in non-athletes, while the weight was higher in athletes than in non-athletes. This testifies the impact of physical activities on the parameters of body composition. Similar studies have identified significant correlations between decreased adipose tissue levels and regular exercise [79,80].

The results of our study confirm and they are in accordance with the results of previous studies that showed a decrease in body fat [81,82] and increased muscle volume [83,84], at different age categories [85,86] and by practicing different types of physical activities [87,88]. Expert recommendations, based on complex studies, suggest that 150 min of moderate aerobic physical activity or 75 min of intense aerobic activity per week may lead to improved cardiovascular health; however, studies have not shown significant decreases in obesity or clinical weight optimization without caloric restrictions as a result of dietary readjustments [36,87,89].

In the present study, magnetic bioresonance and bioimpedance technologies were used for reasons of cost efficiency, time, and easy applicability; the results aimed to identify differences in body composition parameters due to regular physical activity in two samples of healthy sports and non-sports subjects. The studies performed on different categories of populations have shown that, by comparison, the results of computed tomography (CT) and the BIA have statistically significant correlations in terms of assessing visceral obesity and other parameters of body composition. BIA can be used as an alternative to CT as a standard method [90,91,92].

After completing the study, we were able to identify some relevant limitations. The study included only subjects aged between 18 and 25 years, and the extension of the study to other age groups and to totally sedentary people would help to identify particular aspects according to the topic of the current study. Another limitation of the study was the non-identification of the volume and intensity of physical activities performed by the study sample, this aspect may have important implications on the level of physical fitness and implicitly on the parameters of body composition. A relevant limitation of the current study was that the results of the current study have not been compared with the results of other studies in which determinations are made by classical methods of investigating body composition parameters, as well the use of the magnetic bioresonance technology, which is difficult to interpret due to undefined parameters, and is difficult to relate to other studies.

The strengths of the study can be summarized in the complexity of obesity-related parameters analyzed in the study, the large number of subjects included in the study, the use of bioimpedance and magnetic bioresonance technologies to collect the results of the study, we take into consideration, for this study, two categories of subject active and semi-active students from various academic specializations.

## 5. Conclusions

The results of the study highlight significant differences in the correlations of obesity between the samples of active and semi-active sports students, for both sexes. The use of magnetic bioresonance and bioimpedance investigation technologies facilitated the identification of three categories of obesity-related multifactorial parameters, namely the body componential analysis, nourishment and the obesity analysis report.

The biggest differences regarding obesity and body composition were recorded between active and significant sports student samples. For both sexes, the most important parameters were abnormal lipid metabolism coefficient, triglyceride content of abnormal coefficient, degree of body obesity (ODB %), body mass index (BMI), body fat (kg), muscle volume (kg), lean body weight (kg). The results of the study highlight significant differences in all areas, noted between the sample of active athletes compared to those that are semi-active, emphasizing the impact of regular and systematic physical activity on body composition, obesity and health.

This study advances our understanding of the complex impact that physical activities have on multiple parameters of body composition and directly on the health of active students compared to semi-active or sedentary students. Regular exercise to promote active behaviors can have important benefits on body composition and can help optimize body weight and prevent obesity. Further studies are necessary to better understand how bio-resonance and magnetic resonance technologies can be optimally used in clinical practice. The health consequences of obesity can be diminished through physical activity; exercise is an effective means of managing obesity.

## Figures and Tables

**Table 1:** Reference values of the obesity analysis report.

Testing Item	Normal Range	
	Male	Female
Abnormal lipid metabolism coefficient	1.992–3.713	1.992–3.713
Brown adipose tissue abnormalities coefficient	2.791–4.202	2.791–4.202
Hyperinsulinemia coefficient	0.097–0.215	0.097–0.215
Nucleus of the hypothalamus abnormal coefficient	0.332–0.626	0.332–0.626
Triglyceride content of abnormal coefficient	1.341–1.991	1.341–1.991

Other reference values are the body fat percentage of males: 20–25% is overweight, >25% is obesity; the body fat percentage of females: 17–24% is normal, 25–30% is overweight, >30% is obesity; the muscle volume is 35–48% body weight.

**Table 2:** Statistical analysis of the obesity analysis report for the male groups.

Parameters	Groups	X	SD	DX	DDS	CI95% Lower	CI95% Uper	*t*	*p*	S-W
Abnormal lipid metabolism coefficient	Sp	1.574	1.053	−0.798	1.369	−1.091	−0.504	−5.403	0.000	0.711
	Nsp	2.372	0.987							0.802
Brown adipose tissue abnormalities coefficient	Sp	2.952	0.792	−0.218	0.953	−0.422	−0.014	−2.125	0.036	0.867
	Nsp	3.171	0.620							0.776
Hyperinsulinemia coefficient	Sp	0.140	0.039	−0.129	0.494	−0.235	−0.023	−2.431	0.017	0.791
	Nsp	0.270	0.498							0.808
Nucleus of the hypothalamus abnormal coefficient	Sp	0.462	0.082	−0.031	0.137	−0.014	0.061	−2.140	0.035	0.815
	Nsp	0.494	0.101							0.821
Triglyceride content of abnormal coefficient	Sp	3.001	1.095	0.844	1.186	0.590	1.099	6.600	0.000	0.865
	Nsp	2.157	0.602							0.918

Sp—group of sports students, Nsp—group of non-sports students, X—average, SD—standard deviation, DX—average difference, DDS—standard deviation of DX, CI—interval of confidence, *t*—value of Student’s test, *p*—significant level of probability.

**Table 3:** Statistical analysis of the body composition parameters in male participants.

Parameters	Groups	X	SD	DX	DDS	CI95% Lower	CI95% Uper	*t*	*p*	S-W
Obesity degree of body(ODB %)	Sp	103.453	13.582	−10.290	17.164	6.610	13.970	5.560	0.000	0.856
	Nsp	113.744	10.448							0.903
Body mass index (BMI)	Sp	22.275	2.940	−2.326	3.729	1.527	3.126	5.786	0.000	0.876
	Nsp	24.602	2.324							0.819
Body cell mass (BCM)	Sp	23.888	3.644	−1.597	0.518	0.566	2.628	3.082	0.003	0.921
	Nsp	25.485	2.458							0.845

Sp—group of sports students, Nsp—group of non-sports students, X—average, SD—standard deviation, DX—average difference, DDS—standard deviation of DX, CI—interval of confidence, *t*—value of Student’s test, *p*—significant level of probability.

**Table 4:** Statistical analysis of the Nourishment for the male groups.

Parameters	Groups	X	SD	DX	DDS	CI95% Lower	CI95% Uper	*t*	*p*	S-W
Intracellular Fluid (L)	Sp	18.214	1.763	1.150	3.424	0.415	1.884	3.115	0.003	0.823
	Nsp	17.064	2.584							0.795
Extracellular Fluid (L)	Sp	9.327	0.901	0.583	1.764	0.205	0.962	3.068	0.003	0.789
	Nsp	8.7442	1.336							0.769
Protein (kg)	Sp	7.2228	0.696	0.381	1.352	0.091	0.671	2.618	0.010	0.923
	Nsp	6.8409	1.026							0.912
Inorganic substance	Sp	25.861	2.077	−0.300	3.803	−1.116	0.514	−0.733	0.466	0.867
	Nsp	26.162	2.820							0.827
Body fat (kg)	Sp	15.299	4.064	−2.042	5.243	0.918	3.166	3.612	0.001	0.875
	Nsp	17.341	2.893							0.792
Body moisture (kg)	Sp	25.808	3.943	−1.740	5.198	0.626	2.855	3.105	0.003	0.819
	Nsp	27.548	2.650							0.835
Muscle volume (kg)	Sp	35.179	3.801	2.565	6.834	1.100	4.031	3.482	0.001	0.917
	Nsp	32.613	4.975							0.881
Lean body weight (kg)	Sp	56.286	7.402	−2.841	11.302	−5.265	-0.418	−2.332	0.022	0.814
	Nsp	59.127	7.678							0.821
Weight (kg)	Sp	74.186	11.240	−3.790	14.770	0.623	6.957	2.380	0.020	0.827
	Nsp	77.976	7.655							0.831

Sp—group of sports students, Nsp—group of non-sports students, X—average, SD—standard deviation, DX—average difference, DDS—standard deviation of DX, CI—interval of confidence, *t*—value of Student’s test, *p*—significant level of probability.

**Table 5:** Statistical analysis of the obesity analysis report for the female groups.

Parameters	Groups	X	SD	DX	DDS	CI95% Lower	CI95% Uper	*t*	*p*	S-W
Abnormal lipid metabolism coefficient	Sp	1.830	0.952	−1.063	1.194	−1.380	-0.746	−6.721	0.000	0.891
	Nsp	2.894	0.789							0.895
Brown adipose tissue abnormalities coefficient	Sp	2.765	0.943	−0.348	1.047	−0.626	−0.070	−2.509	0.015	0.789
	Nsp	3.114	0.474							0.906
Hyperinsulinemia coefficient	Sp	0.167	0.057	−0.052	.119	−0.083	−0.020	−3.311	0.002	0.918
	Nsp	0.219	0.097							0.913
Nucleus of the hypothalamus abnormal coefficient	Sp	0.438	0.080	−0.073	.117	−0.104	−0.042	−4.749	0.000	0.836
	Nsp	0.512	0.090							0.821
Triglyceride content of abnormal coefficient	Sp	3.118	1.345	0.807	1.395	0.437	1.178	4.371	0.000	0.847
	Nsp	2.310	0.693							0.839

Sp—group of sports students, Nsp—group of non-sports students, X—average, SD—standard deviation, DX—average difference, DDS—standard deviation of DX, CI—interval of confidence, *t*—value of Student’s test, *p*—significant level of probability.

**Table 6:** Statistical analysis of the body composition parameters in female participants.

Parameters	Groups	X	SD	DX	DDS	CI95% Lower	CI95% Uper	*t*	*p*	S-W
Obesity degree of body (ODB %)	Sp	104.140	17.581	−8.082	19.217	2.983	13.181	3.175	0.002	0.911
	Nsp	112.228	10.244							0.848
Body mass index (BMI)	Sp	21.771	3.453	−2.285	3.913	1.247	3.324	4.410	0.000	0.909
	Nsp	24.057	2.475							0.879
Body cell mass (BCM)	Sp	21.196	5.132	−1.591	5.119	0.232	2.949	2.346	0.023	0.815
	Nsp	22.787	1.675							0.845

Sp—group of sports students, Nsp—group of non-sports students, X—average, SD—standard deviation, DX—average difference, DDS—standard deviation of DX, CI—interval of confidence, *t*—value of Student’s test, *p*—significant level of probability.

**Table 7:** Statistical analysis of nourishment for the female groups.

Parameters	Groups	X	SD	DX	DDS	CI95% Lower	CI95% Uper	*t*	*p*	S-W
Intracellular Fluid (L)	Sp	14.588	3.399	−0.346	3.682	−1.323	0.630	−0.710	0.481	0.902
	Nsp	14.935	1.268							0.893
Extracellular Fluid(L)	Sp	7.3953	1.801	−0.324	1.858	−0.817	0.169	−1.316	0.193	0.872
	Nsp	7.7193	.600							0.791
Protein(kg)	Sp	5.6574	1.475	−0.307	1.576	−0.725	0.110	−1.472	0.146	0.810
	Nsp	5.9649	.482							0.823
Inorganic substance	Sp	18.963	3.819	1.296	4.430	0.121	2.472	2.210	0.031	0.871
	Nsp	17.666	2.071							0.849
Body fat (kg)	Sp	14.263	1.716	−2.586	6.335	0.905	4.267	3.082	0.003	0.903
	Nsp	16.849	6.248							0.793
Body moisture (kg)	Sp	22.947	5.436	0.194	5.861	−1.360	1.749	0.251	0.003	0.872
	Nsp	22.752	1.847							0.817
Muscle volume (kg)	Sp	26.434	6.157	−2.570	9.103	0.154	4.985	2.132	0.001	0.824
	Nsp	23.864	5.616							0.871
Lean body weight (kg)	Sp	43.778	6.156	−4.118	11.149	1.160	7.077	2.789	0.007	0.843
	Nsp	47.897	9.322							0.828
Weight (kg)	Sp	65.014	14.37	4.364	15.127	0.351	8.378	2.178	0.034	0.818
	Nsp	60.649	5.862							0.832

Sp—group of sports students, Nsp—group of non-sports students, X—average, SD—standard deviation, DX—average difference, DDS—standard deviation of DX, CI—interval of confidence, *t*—value of Student’s test, *p*—significant level of probability.
